# Genetic Structure of IQ, Phonemic Decoding Skill, and Academic Achievement

**DOI:** 10.3389/fgene.2019.00195

**Published:** 2019-03-18

**Authors:** Nikita K. Lazaroo, Timothy C. Bates, Narelle K. Hansell, Margaret J. Wright, Nicholas G. Martin, Michelle Luciano

**Affiliations:** ^1^Department of Psychology, School of Philosophy, Psychology and Language Sciences, The University of Edinburgh, Edinburgh, United Kingdom; ^2^Queensland Brain Institute, The University of Queensland, Brisbane, QLD, Australia; ^3^Centre for Advanced Imaging, The University of Queensland, Brisbane, QLD, Australia; ^4^Genetic Epidemiology, QIMR Berghofer Medical Research Institute, Brisbane, QLD, Australia

**Keywords:** reading ability, scholastic achievement, multivariate modeling, twin design, intelligence

## Abstract

The aim of this study was to examine whether phonemic decoding skill (deficits of which characterize dyslexia) shares genetic and/or environmental covariance with scholastic abilities independent of general intelligence. Non-word reading ability, verbal and non-verbal IQ, and standardized academic achievement (Queensland Core Skills Test; QCST) were measured in Australian twins (up to 876 twin pairs and 80 singleton twins). Multivariate genetic analysis showed the presence of a general genetic factor, likely reflecting crystallized ability, which accounted for 45–76% of phenotypic variance in QCST scores, 62% of variance in Verbal IQ, 23% of variance in Performance IQ, and 19% of variance in phonological reading ability. The phonemic decoding genetic factor (explaining 48% of variance in phonemic decoding) was negatively associated with mathematical achievement scores (0.4%). Shared effects of common environment did not explain the relationship between reading ability and academic achievement beyond those also influencing IQ. The unique environmental reading factor (accounting for 26% of variance) influenced academic abilities related to written expression. Future research will need to address whether these reading-specific genetic and unique environment relationships arise from causal effects of reading on scholastic abilities, or whether both share a common influence, such as pleiotropic genes/environmental factors.

## Introduction

Success in education has important consequences for later life health, occupation, and even mortality (Ross and Wu, 1995; Miech et al., 2011). Much research has focussed on finding modifiable predictors of educational outcomes. Behavior genetic researchers have contributed to this goal by dissociating the genetic and environmental sources of covariance between these predictors and academic success. This research has established that educational attainment is strongly related to psychometric intelligence, not only phenotypically (e.g., Roth et al., 2015), but also genetically (e.g., Thompson et al., 1991). The genetic correlation between achievement and intelligence is high, ranging from 0.50 to 0.85, depending on age and the type of intelligence test used (Thompson et al., 1991; Wainwright et al., 2005a). This genetic association accounts for the majority (>70%) of the phenotypic correlation between intelligence and achievement and this overlap increases with age (Wainwright et al., 2005b; Kovas et al., 2007).

Recent research has widened the search for traits underlying educational attainment to include, for example, personality and health (De Ridder et al., 2013; Zuffianò et al., 2013; Briley et al., 2014), and within genetically sensitive designs (e.g., Luciano et al., 2006; Rimfeld et al., 2016). Krapohl et al. (2014) found that while intelligence accounted for more heritability in achievement than any individual trait, put together, traits such as self-efficacy, school environment, and health and well-being, accounted for more than 50% of the heritability of academic achievement. Beyond traits like self-efficacy and persistence, multiple domain specific abilities are associated with scholastic achievement (Gustafsson and Balke, 1993; Gathercole et al., 2004). Literacy and reading is, of course paramount among these (Ritchie and Bates, 2013). The purpose of the present study was to test whether a phonological measure linked to reading ability, and thought to form an integral component of reading comprehension (Engen and Høien, 2002), accounts for variance in academic achievement independent of general intelligence and, if so, whether this association is environmental or genetic in origin.

Phonological reading ability, or decoding, is the ability to detect, retrieve and apply the sound elements of one’s language to written text (Wagner and Torgesen, 1987; de Jong and van der Leij, 2002). Prominent models of reading development emphasize the importance of phonological decoding in both single word reading acquisition and reading comprehension (e.g., Hoover and Gough, 1990; Coltheart et al., 1993; Ehri et al., 2001). Behavioral studies of early reading development show that phonological decoding abilities predict unique variance in subsequent reading comprehension, above and beyond the variance accounted for by other linguistic abilities like vocabulary and listening comprehension (de Jong and van der Leij, 2002; Oakhill and Cain, 2012).

Multivariate analyses of the genetic structure of phonological decoding suggest that decoding shares variance with both single word recognition and complex reading comprehension. For example, phonological awareness in pre-school aged children is influenced by two genetic factors; an additive genetic factor shared with print knowledge, which predicts single word reading ability until late elementary school (Byrne et al., 2005, 2009; *see also*, Wagner et al., 1997; Lonigan et al., 2000), and a specific additive genetic factor which predicts Grade 2 reading comprehension (Byrne et al., 2009). In 8- to 18-year-olds, phonological decoding shares one genetic factor with orthographic decoding, verbal intelligence (VIQ) and phoneme awareness, and a second genetic factor with phoneme awareness and word recognition (Gayán and Olson, 2003). After variance accounted for by these two genetic factors, phonological decoding had no specific genetic effects. Phonological decoding, therefore, shows unique effects specific to phonological abilities (Bates et al., 2007) and shares genetic effects with general comprehension abilities, including VIQ. One model, compatible with these data, would be that acquiring reading skills could improve reading comprehension or general ability (Ritchie et al., 2015).

This proposed causal relationship in which improvements in phonological skills cause increases in reading comprehension, if true, would have important consequences for academic achievement. Poor reading comprehension in childhood predicts poorer verbal abilities and general knowledge in late adolescence (Cunningham and Stanovich, 1997; Mol and Bus, 2011). Cunningham and Stanovich (1997) found that print exposure, a proxy measure for reading frequency, accounted for most of this relationship. Further, print exposure (measured by an author recognition test) shared overlapping genetic and environment variance with reading and spelling skill and verbal IQ (Martin et al., 2009). Out-of-school reading and print exposure facilitate the acquisition of knowledge in many verbal and cognitive domains, making reading an important method for learning concepts beyond what is taught in a school curriculum (Echols et al., 1996; Mol and Bus, 2011; Ritchie et al., 2015).

Turning to associations of reading comprehension with school performance, there is evidence that poor comprehension presents a barrier to pupil’s ability to understand and integrate material taught in the classroom. Logan et al. (2011) demonstrated that poor reading comprehension could be explained by poor phonological decoding ability and a lack of intrinsic motivation. Poor phonological decoding ability is likely to slow student’s acquisition of an efficient reading system, thus slowing their ability to derive meaning from written materials. Second, a lack of motivation implies students will be less likely to practice and improve their reading skills, resulting, again, in poorer academic performance (Butkowsky and Willows, 1980; Morgan and Fuchs, 2007; Haimovitz et al., 2011).

Relatively few studies have investigated the genetic and environmental relationships between phonemic decoding skill and academic achievement. This is important, because apparently causal relationships, even ones with great intuitive plausibility, such as the association of parental communication with children and children’s own language and reading development can be confounded by genetic transmission (Puglisi et al., 2017). Among genetically informed studies, those which consider reading ability tend to use global measures of reading comprehension and focus on mathematical achievement. For example, Hart et al. (2009) studied 12-year-old children and found that measures of reading comprehension and word decoding accounted for all of the genetic variance in mathematical problem solving. Similarly, other studies which use global measures of reading ability find high genetic overlap between reading and mathematical achievement (Thompson et al., 1991; Kovas et al., 2005; Markowitz et al., 2005). It is impossible to determine from these studies, however, which aspects of reading ability influence variance in academic achievement.

In a paper which forms the basis of our hypotheses in the present paper, Light, DeFries and Olson (1998) demonstrated that most of the genetic variance in VIQ, phonological decoding, global reading comprehension, and mathematical ability was accounted for by a shared genetic factor. Phonological decoding, however, accounted for significant additional variance in comprehension and mathematical ability. Here, we extend Light et al. (1998) findings by examining whether phonemic decoding skill accounts for unique variance in other academic outcomes beyond mathematics.

Most behavioral-genetic studies on academic achievement or reading ability have examined twins during early childhood (e.g., Thompson et al., 1991; Wadsworth et al., 1995; Bartels et al., 2002b; Kovas et al., 2005). Here, we focus on twins whose academic achievement was measured in their final years of secondary school with the Queensland Core Skills Test (QCST). The QCST is a standardized test of achievement sat by 85% of final year students in Queensland, Australia (Wainwright et al., 2005a). It has some strengths over other measures of academic achievement because it is specifically designed to measure a broad range of higher-order scholastic skills and is not based on a particular curriculum of studies (Viviani, 1990; Matters and Gray, 1994). Unlike research which uses school grades or marks on standardized tests, the QCST evaluates how students apply academic skills across a diverse range of stimuli (Viviani, 1990).

Previous work by Wainwright et al. (2005b), based on a subset of the present study’s sample, found that a general genetic factor shared with IQ accounted for 32–73% of phenotypic variance in the five academic factors of the QCST. Two subtests of the QCST, Create and Present, which measures written fluency, and Apply Techniques and Procedures, which measures mathematical problem solving, also had significant specific genetic effects. The Comprehend and Collect subtest, which assesses comprehension and manipulation of verbal and non-verbal stimuli, demonstrated evidence for specific genetic effects, although these did not reach significance. The final two subtests of the QCST, Structure and Sequence and Analyze, Assess and Conclude had a genetic correlation of 1.00 with all genetic variance in these subtests being accounted for by genetic effects shared with VIQ and PIQ. Thus, although representing conceptually different subtests, Structure and Sequence; Analyse, Assess and Conclude appear to be evaluating the same higher-order cognitive abilities assessed by intelligence tests. In the present study, therefore, we analyzed only the Create and Present, Comprehend and Collect, and Apply Techniques and Procedures factors of the QCST.

Consistent with previous studies (e.g., Kovas et al., 2005; Wainwright et al., 2005b), our first hypothesis was that a general genetic factor (general intelligence) would influence variance in all traits. Our second hypothesis was that a group genetic factor would be required to account for variance in measures associated specifically with reading ability (phonemic decoding skill, Comprehend and Collect, Create and Present and Apply Techniques and Procedures subtests). This follows from findings of specific genes influencing reading skill (e.g., Light et al., 1998; Gayán and Olson, 2003), and which we predict share additional variance with academic achievement beyond general cognitive ability. Fourth, we hypothesized that a single shared environment factor would be required, and would be sufficient to account for common environmental variation in the data, as has been found previously (Eaves et al., 1984). More speculatively, we conjectured that task-specific genetic factors would be required for good model fit. No specific hypotheses were made regarding the contribution of unique environmental effects to variance in each of the variables.

## Materials and Methods

### Participants

Data were collected from twins in the Brisbane Memory, Attention and Problem Solving (MAPS) study, the 16-year-old wave of the Brisbane Longitudinal Twin Study (Wright et al., 2001; Wright and Martin, 2004). Data for this wave was collected between 1996 and 2013. Results here are reported for a maximal sample of 876 twin pairs (300 MZ, 576 DZ) and for one twin from a further 80 pairs (the cotwin excluded or missing for various reasons). Data were not available for all twins on all measures. VIQ data were available from 664 twin pairs and 64 individual twins. PIQ data were available for 665 twin pairs and 63 individual twins. Participants were IQ tested as close as possible to their sixteenth birthday (16.2 years, ±1.27 *SD*, range 15–28, 4% tested at 18 years or older). Phonological reading data were available for 876 twin pairs and 80 individual twins (17.39 years, ±3.20 *SD*, range 12–25). Data collection for the reading test happened over a longer period of time; some participants took the reading test before IQ testing (*n* = 1096), contemporaneous with IQ testing (*n* = 521), or after IQ testing (*n* = 1337). QCST data were available for 470 twin pairs and 155 individual twins (81% of sample aged 17, range 16–20) with reading data. QCST data could be missing because (1) participants had left school prior to administration of the test; (2) only participants wishing to apply for university were required to sit the QCST in their final year of school, (3) consent had not been obtained to access their QCST results, and (4) their information could not be successfully matched with that held by the Queensland Curriculum and Assessment Authority (QCAA).

Zygosity for same-sex twin pairs was determined using the AmpFISTR Profiler Plus Amplification kit, ABI, which examines 10 independent DNA markers; nine short tandem repeat (STR) loci and one homologous region on the X and Y chromosomes (Wright and Martin, 2004; Wainwright et al., 2005a). Twin pairs were checked for concordance across the nine STR loci and results were compared against blood group results and/or phenotypic data such as hair, skin and eye color. Probability of error in assigning zygosity was less than 10^-4^. Subsequently, zygosity was confirmed when all twins were genotyped on SNP arrays for genome-wide association studies.

Informed consent was obtained from participants and their parents, if under the age of 18, prior to any testing. Participants were excluded if parental report indicated that either twin had a history of significant head injury, neurological or psychiatric illness, substance abuse or dependence, or current use of medication with known effects on the central nervous system. All participants had normal or corrected-to-normal vision. The MAPS and Brisbane Longitudinal Twin studies were approved by the QIMR Berghofer Human Research Ethics Committee.

### Measures and Procedure

#### Multidimensional Aptitude Battery (MAB)

The MAB is a multiple-choice test of general intelligence based on the revised Wechsler Adult Intelligence Scale (WAIS-R) (Wechsler, 1981; Jackson, 1984). It is designed for cognitive assessments of adults and adolescents aged 16 and older. Five subtests were administered: three assessing VIQ (Vocabulary, Information, Arithmetic) and two assessing PIQ (Spatial, Object Assembly). Correlations between the MAB and WAIS-R verbal and performance subscales are 0.94 and 0.79, respectively (Jackson, 1984). A previous study using a subsample of the twins used here reported test-retest reliabilities of 0.89 for VIQ and 0.97 for PIQ (Luciano et al., 2001). Participants were administered the MAB during an in-person assessment in the laboratory. Further details on the experimental procedure for IQ testing can be found in other papers from the MAPS study (e.g., Luciano et al., 2001; Wright and Martin, 2004).

#### Queensland Core Skills Test (QCST)

The QCST is a standardized test of academic achievement which must be sat by all year 12 students hoping to enter tertiary education. The QCST assesses students on higher-order scholastic skills that are taught in a wide range of courses. Such skills include synthesizing and interpreting data, mathematical problem solving, comprehending and explaining written text, perceiving complex patterns, performing exact and approximate numerical calculations, understanding mechanical-spatial relationships, producing written prose and interpreting themes within visual and written stimuli (Queensland Curriculum and Assessment Authority [QCAA], 2018c). As the QCST assesses scholastic skills rather than subject-specific knowledge, it limits any advantage arising from having studied a particular curriculum at school (Matters and Gray, 1994). Historical data from 1992 to 2007 showed the QCST correlated 0.70–0.75 with a within-school ranking of students and with actual marks attained in school (Queensland Studies Authority, 2006, 2007, 2008).

The QCST is composed of four exam papers; a written task, two multiple choice papers and a collection of short response questions. Students complete these papers over two consecutive days during the third term of their final year (Queensland Curriculum and Assessment Authority [QCAA], 2018b). The content of the exam falls within five predetermined academic factors. The Comprehend and Collect factor assesses the students’ ability to comprehend, manipulate and interpret data from a wide range of stimuli, including written, mathematical and visual stimuli. SS assesses the ability to select, sort and organize information and detect complex patterns and relationships. Analyse, Assess and Conclude involves induction and deduction of relationships, evaluating the merit of text and drawing conclusions from text. Create and Present assesses a students’ ability to use and structure written language to effectively communicate ideas. Finally, Apply Techniques and Procedures involves making calculations and mathematical problem solving (Queensland Curriculum and Assessment Authority [QCAA], 2018a,b). Based on previous results, only Comprehend and Collect, Create and Present, and Apply Techniques and Procedures factors will be considered in this study (Wainwright et al., 2005b). More information regarding the design and administration of the QCST can be found in Wainwright et al. (2005a) and on the QCAA Website^1^.

Written permission for obtaining QCST results was provided by participants and their guardians and forwarded to the QCAA, previously the Queensland Studies Authority. A database containing the total score for the QCST, scores for each examination paper and scores for each of the five QCST factors were returned along with the year in which the QCST was completed. All identifying information contained in the database was removed immediately upon receipt. Annual statistics, including means, standard deviations and score ranges were also provided by the QCAA.

Scores for each of the five factors are calculated based on performance on relevant questions across the four examination papers. The maximum score attainable for each factor varies according to year; therefore, scores for the current sample were standardized using the means and *SD* of the entire Queensland sample within each year. Standardized data from 1996 to 2011 were analyzed together.

#### Non-word Reading Test

Non-word reading was assessed using an extended version of Castles and Coltheart’s test of non-word reading (Castles and Coltheart, 1993). Additional items were added to make the test more suitable for older participants (Bates et al., 2004). Non-word reading is a reliable and valid measure of phonological decoding (Campbell et al., 1997; Bates et al., 2004). Castles and Coltheart (1993) test of non-word reading has also been validated by other phonological measures such as phonological choice and phoneme deletion tasks (Olson et al., 1994; Castles et al., 1999).

The non-word reading test was administered over the phone; as such, a small number of answers were not recorded due to phone errors. Test scores were calculated as a ratio of correctly answered items to total number of recorded scores. Further information on experimental procedures can be found elsewhere (e.g., Bates et al., 2004; Wright and Martin, 2004).

### Statistical Analyses

#### Model-Fitting

Structural equation modeling techniques were used to partition the observed phenotypic variance in each variable into three components: additive genetic (A), shared environmental (C), and non-shared environmental (E). Additive genetic influences represent the sum of the additive effects of alleles at all gene loci that influence a trait. Shared environmental influences are non-genetic sources of variance that contribute to the similarity of twins in a pair, for instance socioeconomic status, parenting style and common peer or school influences. Non-shared, or unique, environmental influences are factors that contribute to differences within a twin pair, such as differential treatment by parents, teachers and peers. The unique environmental factor also includes measurement error. Twin data enable these three sources of variance to be estimated because monozygotic (MZ) and dizygotic (DZ) twins differ in the proportion of genes shared within a pair. MZ twins share 100% of their segregating genes while DZ twins share on average 50% of their segregating genes. As such, the A factor correlates 1 between MZ twins and 0.5 between DZ twins. Both MZ and DZ pairs correlate 1 for the common environment factor (Rijsdijk and Sham, 2002).

Multivariate Cholesky ACE decompositions with all variables included in the model were then conducted to estimate the extent to which each variance component contributed to covariation between variables. The fit of the hypothesized model was compared against the full Cholesky decomposition.

Analyses were conducted using full-information maximum-likelihood (ML) procedures on raw data with the OpenMX and umx packages in R (Neale et al., 2016; Bates et al., 2017). Analysis of raw data increases the accuracy of means and variances estimates, thus improving the accuracy of covariance estimates during model estimation (e.g., Harlaar et al., 2005). ML procedures were chosen as they are best able to handle missing data (Enders and Bandalos, 2001). Model fit was tested by examining differences in -2 log-likelihood (-2LL) between nested models, which is distributed as a χ^2^ statistic. A significant χ^2^ value was interpreted as a loss of model fit. The full analysis strategy is laid out in order below.

#### Data Screening

QCST data were standardized and data for all variables were screened for normality and outliers. Age-residualised standard scores were used for genetic analysis where age effects were significant. Outliers falling 3.5 *SD* from the mean were Winsorized to the ±3.5 *SD* value. Unless otherwise stated, a significance level of 0.05 was used.

#### Representativeness of Sample

To determine if the sample was representative of the population of QCST test-takers, the mean and *SD* of the standardized Comprehend and Collect, Create and Present and Apply Techniques and Procedures factor scores were constrained to zero and one, respectively. Constrained models were then compared to an unconstrained model. A significant χ^2^ value was interpreted as the constrained model being unlikely to be true.

#### Testing Equality of Means, Variances, and Covariances According to Zygosity and Sex

To determine regularity in sampling, the equalities of means and variances according to birth order and zygosity [MZF, MZ females; MZM, MZ males; DZF, DZ females; DZM, DZ males and opposite-sex twin pairs with the female (DZFM) or male (DZMF) born first] for each variable were tested. Increasingly restrictive nested models were assessed, with each model being compared to the preceding, less restrictive model. A significant change in -2LL value was interpreted as a given constraint hypothesis being unlikely to be true.

The equality of covariances between MZF and MZM and between DZF and DZM twin pairs was tested to assess the presence of scalar sex limitation: differences in the magnitude of genetic effects on each variable across sex. Additionally, the equality of covariances between same-sex DZ (DZF, DZM) pairs and opposite-sex DZ (DZFM, DZMF) pairs was tested to determine whether different genes were being expressed according to sex (non-scalar sex limitation) (Neale and Cardon, 1992).

#### Statistical Analysis

**Figure 1:**
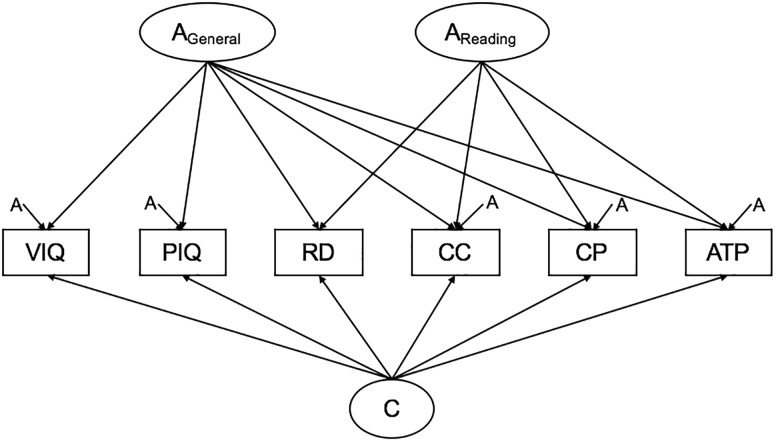
Path diagram of hypothesized model. Note: No specific hypotheses are made regarding the unique environmental structure. These paths are omitted for clarity. VIQ, verbal IQ; PIQ, performance IQ; RD, reading; Comprehend and Collect, comprehend and collect; CP, create and present; ATP, apply techniques and procedures.

A full ACE Cholesky decomposition was first conducted with all variables, it specifies as many A, C, and E factors as there are variables, with each set of factors loading on one less variable than the previous set. A model with one general genetic factor (A_General_), one group genetic factor loading on reading ability and the QCST subtests (A_Reading_), specific genetic factors, a general common environment factor and unique environmental influences (Figure 1) was compared to the full Cholesky decomposition to test the hypothesis that reading ability shares genetic covariance with areas of academic achievement independent of IQ. The R script used to run this analysis can be found in the Supplementary Material.

## Results

### Data Screening

Data for all variables were visually inspected for normality. Two participants were given VIQ or PIQ scores of 0 because of software failure and were considered missing. Reading data were negatively skewed and transformed by a reflected logarithmic function. Outlying values were observed for 2 VIQ scores, 2 Create and Present scores, and 1 reading score; these values were Winsorized and normality was re-inspected.

### Representativeness of Sample

Means for Comprehend and Collect (+0.22 *SD*), Create and Present (+0.19 *SD*) and Apply Techniques and Procedures(+0.18 *SD*) could not be constrained to the population value of zero (Comprehend and Collect: χ^2^ = 56.79, *df* = 1, *p* < 0.05; Create and Present: χ^2^ = 45.36, *df* = 1, *p* < 0.05; Apply Techniques and Procedures: χ^2^ = 33.01, *df* = 1, *p* < 0.05). That is, the sample of participants used here is slightly more successful in these tests than the total population of test takers. The variances for Comprehend and Collect and Apply Techniques and Procedures could be constrained to one without a significant loss of fit. The variance for Create and Present (0.87) could not be constrained to one (Create and Present: χ^2^ = 9.74, *df* = 1, *p* < 0.05); its smaller variance was in line with the sample being selected for higher ability.

### Equality of Means, Variances, and Covariances According to Zygosity and Sex

Table 1 reports the mean and *SD* for each variable (age-residualised phonological reading, Apply Techniques and Procedures, Comprehend and Collect and Create and Present) separately for males and females. When testing for equality of means, variances and covariances, a Bonferroni correction was applied because the mean, variance, and covariance of each variable was compared 12 times (Bonferroni corrected *p*-value = 0.004). Means and variances could be equated across birth order and zygosity for all variables. Means could not be equated across sex for VIQ, PIQ, Create and Present and Apply Techniques and Procedures. Males performed better than females on the VIQ, PIQ and Apply Techniques and Procedures tests. Females performed better than males on the Create and Present test. Means were therefore not equated across sex for VIQ, PIQ, Create and Present, and Apply Techniques and Procedures in subsequent analyses. Variances could be equated across sex for all variables.

**Table 1:** Mean and standard deviation of variables for females and males, and twin correlations according to zygosity.

	Means (standard deviations)				Correlations						
	Females		Males		MZF	MZM	DZF	DZM	DZOF	DZOM
			
	n	M(*SD*)	n	M(*SD*)	(90–160)	(65–140)	(103–150)	(71–151)	(68–130)	(73–145)
VIQ	746	108.50 (10.81)	646	112.00 (11.48)	0.81	0.80	0.48	0.53	0.59	0.41
PIQ	746	109.75 (16.09)	647	114.57 (15.74)	0.82	0.79	0.40	0.53	0.35	0.22
RD	937	-0.89 (0.34)	895	-0.94 (0.36)	0.72	0.78	0.31	0.50	0.40	0.36
CC	618	-0.02 (0.98)	477	0.02 (1.03)	0.75	0.75	0.35	0.34	0.38	0.43
CP	618	0.11 (0.94)	477	-0.14 (0.98)	0.47	0.51	0.28	0.39	0.18	0.34
ATP	618	-0.19 (0.97)	477	0.25 (0.98)	0.73	0.73	0.37	0.34	0.36	0.42

There was no evidence for scalar or non-scalar sex limitation in any of the variables given the equality of correlations for same-sex and opposite-sex DZ twin pairs. Covariances could be equated across MZ and DZ twin pairs without a loss of model fit for Create and Present, indicating the presence of higher common environmental variance in this variable. Results from the full and nested sub-models can be found in Supplementary Table S1.

### Results

Correlations according to zygosity are shown in Table 1. The greater magnitude of MZ correlations suggests genetic influences for each measure. Phenotypic correlations between the variables are presented in Table 2. The strongest inter-correlations were among the academic achievement measures and VIQ.

**Table 2:** Phenotypic correlations with 95% confidence intervals (ML) among study variables.

	VIQ	PIQ	Reading	CC	CP
VIQ					
PIQ	0.50 (0.46–0.54)				
Reading	0.49 (0.45–0.53)	0.27 (0.24–0.31)			
CC	0.69 (0.66–0.72)	0.47 (0.42–0.51)	0.39 (0.33–0.44)		
CP	0.57 (0.53–0.61)	0.34 (0.30–0.39)	0.40 (0.35–0.45)	0.58 (0.54–0.62)	
ATP	0.70 (0.67–0.73)	0.55 (0.51–0.59)	0.33 (0.27–0.38)	0.69 (0.66–0.72)	0.50 (0.47–0.54)

**Figure 2:**
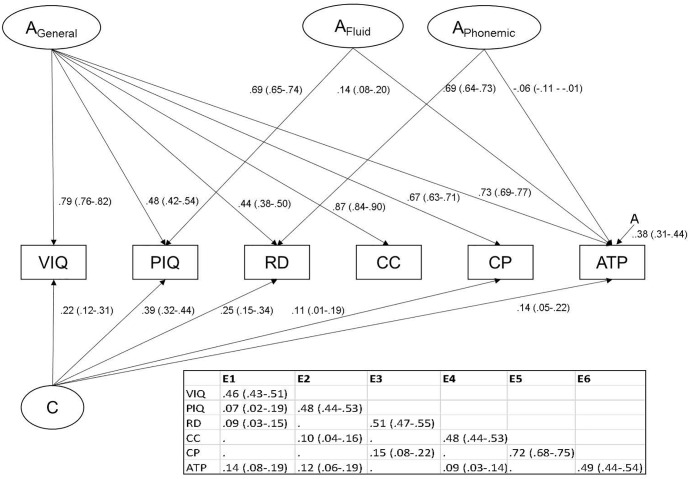
Path diagram showing significant standardized loadings with 95% confidence intervals (ML) of final reduced model. A_General_ represents general additive genetic effects, A_Fluid_ represents fluid ability genetic effects, A_Phonemic_ represents phonological reading additive genetic effects. A represents specific additive genetic effects. The unique environmental (E) structure is presented in the table. VIQ, verbal IQ; PIQ, performance IQ; RD, reading; CC, comprehend and collect; CP, create and present; ATP, apply techniques and procedures.

The hypothesized ACE factor model (Figure 1) resulted in a significant loss of fit compared to the ACE Cholesky decomposition (*p* < 0.0001; see Table 3). The ACE Cholesky decomposition was reduced to arrive at the most parsimonious model of the data. Non-significant paths (as indicated from the 95% CIs; see Supplementary Table S2 for full model paths) were dropped progressively from the additive genetic structure, common environmental structure, then the unique environmental structure. This final model is presented in Figure 2 with standardized path estimates and 95% ML confidence intervals.

**Table 3:** Fit statistics for the full ACE Cholesky decomposition and hypothesized model.

Model	-2LL	df	Δ-2LL	Δdf	p	AIC
Full ACE Cholesky	28925.99	7829				13267.99
Hypothesized model	30260.03	7850	1334.05	21	<0.001	14560.03
Simplified Cholesky	28964.57	7863	38.58	34	0.27	13238.57

*Note: Δ*-2LL* corresponds to a χ^2^ value*.

The general additive genetic factor (A_General_) showed the largest loadings on Comprehend and Collect, VIQ, Apply Techniques and Procedures, then Create and Present, with moderate loadings on PIQ and phonological reading. The A_Phonemic_ factor had a small but significant negative loading on the Apply Techniques and Procedures test (i.e., genes which contribute to better phonemic decoding ability contribute to lower performance on the Apply Techniques and Procedures test). The A_Fluid_ factor (strongly loading on PIQ) had a small influence on Apply Techniques and Procedures. A genetic factor specific to Apply Techniques and Procedures was also present. The common environment factor (C) significantly influenced VIQ, PIQ, phonemic decoding, Create and Present, and Apply Techniques and Procedures. Moderate unique environmental effects (but strong for Create and Present) were supported for each variable, and these included overlapping E effects.

Genetic correlations (beneath diagonal), heritabilities (on diagonal) and the proportion of phenotypic correlation accounted for by genetic effects (above diagonal) are shown in Table 4. Heritabilities for each of the variables were high. Genetic correlations between all variables are high, with the genes for VIQ, Comprehend and Collect and Create and Present overlapping completely. The weakest genetic correlation was between PIQ and phonemic decoding (0.31). In contrast, unique environmental correlations ranged 0.03 (PIQ-Phonemic decoding) to 0.27 (PIQ-Apply Techniques and Procedures). Genetic influences accounted for the majority of the phenotypic correlations between variables. Notably, 89–100% of the phenotypic correlations between Comprehend and Collect and the other variables could be accounted for by genetic effects. Genetic mediation of the phenotypic relationships with reading were the lowest, but nevertheless strong (72% or larger).

**Table 4:** Genetic correlations, heritabilities, and proportions of phenotypic correlations accounted for by genetic factors with 95% confidence intervals.

	*VIQ*	*PIQ*	*READING*	*CC*	*CP*	*ATP*
VIQ	**0.63 (0.58–0.68)**	0.76 (0.68–0.84)	0.72 (0.62–0.80)	1.00 (1–1)	0.93 (0.86–0.99)	0.83 (0.77–0.88)
PIQ	0.57 (0.50–0.62)	**0.71 (0.64–0.77)**	0.78 (0.66–0.88)	0.89 (0.82–0.96)	0.93 (0.86–0.99)	0.82 (0.73–0.89)
RD	0.54 (0.48–0.60)	0.31 (0.26–0.36)	**0.67 (0.60–0.73)**	1.00 (1–1)	0.76 (0.71–0.80)	0.85 (0.77–0.92)
CC	1.00 (1–1)	0.57 (50–0.62)	0.54 (0.48–0.60)	**0.76 (0.71–0.80)**	1.00 (1–1)	0.92 (0.87–0.96)
CP	1.00 (1–1)	0.57 (0.50–0.62)	0.54 (0.48–0.60)	1.00 (1–1)	**0.45 (0.39–0.51)**	0.97 (0.93–1)
ATP	0.87 (0.83–0.91)	0.63 (0.56–0.70)	0.41 (0.33–0.49)	0.87 (0.83–0.91)	0.87 (0.83–0.91)	**0.70 (0.64–0.75)**

*Note: Genetic correlations appear below the diagonal, heritabilities appear in bold on the diagonal and proportions of phenotypic correlations accounted for by genetic effects appear above the diagonal. 95% confidence intervals (ML) presented in parentheses. VIQ, verbal IQ; PIQ, performance IQ; RD, reading; CC, comprehend and collect; CP, create and present; ATP, apply techniques and procedures*.

## Discussion

The primary purpose of this study was to determine if phonemic decoding skill accounts for genetic variance in academic achievement independently of genetic factors accounting for general intelligence. The model that fit best was one with a general genetic factor (A_General_); a second genetic factor (A_Phonemic_) influencing phonemic decoding and also the Apply Techniques and Procedures subtest. Contrary to prediction, a third genetic factor (A_Fluid_) influencing PIQ and Apply Techniques and Procedures, and a fourth genetic factor specific to Apply Techniques and Procedures were required for a well-fitting model. Supporting our hypothesis that a single common environment factor would account for shared environmental effects, a C factor loading on all tests except Comprehend and Collect was required for good fit. Unique environmental effects were mostly overlapping.

Heritabilities for each of the variables were high, and consistent with previous literature (Bartels et al., 2002a,b; Bates et al., 2004; Wainwright et al., 2005b). Confirming much previous research (e.g., Light et al., 1998; Plomin and Kovas, 2005; Wainwright et al., 2005a,b), a general genetic factor accounted for a significant amount of variance in all cognitive tests, but especially academic achievement and VIQ. This pattern of loadings suggests that the general factor extracted was very much related to crystallized learning, or acculturalised knowledge. Again supporting previous findings (e.g., Brooks et al., 1990; Alarcón and DeFries, 1997; Wainwright et al., 2004), phonemic decoding skill was moderately influenced by the general factor, but a larger amount of its genetic variance was explained by the phonemic decoding factor.

A novel finding was that the genetic influences on the phonemic decoding factor negatively influenced Apply Techniques and Procedures, albeit explaining only 0.4% of variance. Our sample was older than previous studies of reading and mathematical ability where positive genetic relations were found (e.g., Kovas et al., 2005; Hart et al., 2009). The QCAA requires all students to reach a satisfactory level of literacy and numeracy in order to achieve the Queensland Certificate of Education, however, senior students are given relative freedom in choosing their course options (Queensland Curriculum and Assessment Authority [QCAA], 2017). It is possible that those students who are stronger readers choose more classes with a literacy versus mathematical component. Nordström et al. (2016) found that better word decoding in students was associated with an increased likelihood of choosing English classes over mathematics classes. Conversely, children with poor reading abilities demonstrate a lower motivation to read, and a low self-concept of their ability (Butkowsky and Willows, 1980; Logan et al., 2011). Nurmi and Aunola (2005) found that in primary and early secondary years, students with a low self-concept of their reading ability were more likely to enroll and succeed in mathematics courses. Thus, the genetic influences which cause early success or failure in reading ability may also motivate students to engage in either reading or mathematics courses. We are cautious to point out, though, that this effect might not generalize to the lower end of the ability distribution given that our sample all had satisfactory levels of literacy and numeracy.

Genetic effects accounted for the majority of phenotypic correlations between variables (72–100%). The lower range of genetic mediation was for relationships with phonemic decoding. Environmental predictors of reading difficulties might therefore also be predictive of correlated academic performance. Unique environmental effects were largely test-specific but did show some overlap with other cognitive skills; unique environmental effects on Create and Present were particularly large (52% of variance compared to, at most, 26% for the others). The unique environmental factor with the largest influence on phonemic decoding influenced Create and Present, but not the other academic skills. Thus, while important for creative writing (including spelling, grammar, punctuation), these unique experiences that facilitate phonological skill do not influence one’s ability to comprehend facts and meaning, nor problem solve or use calculations.

The present study had several limitations. The sample used in this study were above the Queensland average in their performance on the QCST and showed a restricted range of QCST scores at the higher end. Conclusions drawn from these results, therefore, may not be generalize to people in the lower-end of the distribution in academic achievement, reading or intelligence. However, Wainwright et al. (2005a), who reported on a subsample (83%) of the present study found that adjustment for truncate selection had little effect on QCST heritability estimates. The potential for socio-economic status (SES) to moderate the genetic and environmental contributions to phonemic decoding and its covariance with academic skills should be recognized given that the heritability of IQ varies by SES in numerous countries (Tucker-Drob and Bates, 2016). Although in our Australian cohort, for full scale IQ, there is no evidence of gene by SES interaction (Bates et al., 2016). Given that assortative mating has been observed for intelligence and academic achievement, it is also possible that the small effects of common environment reported here may reflect effects of assortative mating increasing the DZ correlation, rather than shared environmental influences (Wainwright et al., 2005a). Finally, while our hypothesized model was based on a causal direction from phonemic decoding to other academic skills, for some subjects, reading was assessed after they took the IQ and/or QCST tests. However, a sensitivity analysis in which we excluded all twin pairs where reading was not collected before IQ/QCTS assessment (i.e., half the sample) showed that the same ACE model fitted the data (these results are available on request).

## Conclusion

In sum, it was hypothesized that phonemic decoding skill would share significant genetic covariance with all tests of achievement. For example, genetic risk of reading difficulties would present a barrier to learning other academic skills and this would be reflected in the genetic covariance independent of IQ. However, this was not supported, indeed, the reverse was found for Apply Techniques and Procedures. With regard to environmental causes of reading variation, the only academic skill that showed unique environment overlap was Create and Present. In conclusion, our findings show that the genes specifically influencing a core reading skill have opposite effects on mathematical/analytic skills; and that unique environmental influences specific to reading positively affect written language proficiency. Future studies are required to establish whether these relationships are the result of pleiotropic effects or a causal effect from phonemic decoding to academic skill.

## Ethics Statement

This study was carried out in accordance with the recommendations of The Australian Code for the Responsible Conduct of Research (predecessor guidelines to the 2018 document), ARC and NHMRC, with written informed consent from all subjects. All subjects gave written informed consent in accordance with the Declaration of Helsinki. The protocol was approved by the QIMR Berghofer Human Research Ethics Committee.

## Author Contributions

NL performed the statistical analyses and drafted the manuscript. ML supervised the study and revised the draft manuscript. NH was involved in data collection and processing. MW, TB, and NM managed the data collection. All the authors commented on the draft manuscript.

## Conflict of Interest Statement

The authors declare that the research was conducted in the absence of any commercial or financial relationships that could be construed as a potential conflict of interest.

## Footnotes

1 https://www.qcaa.qld.edu.au/senior/qcs-test

## Supplementary Material

TABLE S1 Model fitting results from testing the equality of means, variances and covariances for each variable.

TABLE S2 Full Cholesky decomposition standardized parameter estimates with 95% confidence intervals.

DATA SHEET S1 R script to run multivariate analysis.
