# Supplementary material for: Genetic Structure of IQ, Phonemic Decoding Skill, and Academic Achievement
Source: Front Genet. 2019 Mar 18;10:195. doi: 10.3389/fgene.2019.00195 (PMC6436069; doi:10.3389/fgene.2019.00195)
Supplement: TABLE S1 — Model fitting results from testing the equality of means, variances and covariances for each variable. [file Table_1.pdf]

SUPPLEMENTARY MATERIAL

Supplementary Table 1: Model fitting results from testing the equality of means, variances and covariances for each variable

		χ^2 Values					
Hypotheses	Δ *df*	*VIQ*	*PIQ*	*RD*	*CC*	*CP*	*ATP*
H_0		65.94	58.85	77.37	81.73	82.46	57.95
$H1_M$	4	3.43	9.46	6.38	2.67	1.13	0.37
$H2_M$	2	2.16	0.77	0.99	0.34	1.79	0.26
$H3_M$	4	10.54[a]	5.34	4.71	6.79	3.82	2.78
$H4_M$	1	31.11[b]	30.46[b]	3.35	0.10	17.36[b]	45.92[b]
$H1_V$	4	1.77	2.43	3.15	5.92	7.85	0.17
$H2_V$	2	0.05	0.85	1.17	0.09	1.13	0.41
$H3_V$	4	6.59	3.64	5.61	0.26	1.31	2.43
$H4_V$	1	0.16	0.65	2.07	1.02	5.29[a]	0.00
$H1_C$	3	6.19	2.30	3.90	0.51	1.45	0.37
$H2_C$	1	0.01	4.80[a]	0.13	0.60	0.19	0.33
$H3_C$	1	59.12[b]	72.77[b]	64.82[b]	40.48[b]	6.69[a]	29.45[b]
$H3_C$	1	288.46[b]	206.21[b]	280.15[b]	132.81[b]	71.27[b]	137.18[b]

[a] $p < .05$; [b] $p < .004$ (Bonferroni corrected p-value)
