# Supplementary material for: Genetic Structure of IQ, Phonemic Decoding Skill, and Academic Achievement
Source: Front Genet. 2019 Mar 18;10:195. doi: 10.3389/fgene.2019.00195 (PMC6436069; doi:10.3389/fgene.2019.00195)
Supplement: DATA SHEET S1 — R script to run multivariate analysis. [file Data_Sheet_1.pdf]

```
# MULTIVARIATE SCRIPT USED IN LAZAROO ET AL 2019 FRONTIERS IN GENETICS

require(umx)
require(OpenMx)

#data objects
dataMZF <- mxData(observed = MZF_data, type = "raw")
dataMZM <- mxData(observed = MZM_data, type = "raw")
dataDZF <- mxData(observed = DZF_data, type = "raw")
dataDZM <- mxData(observed = DZM_data, type = "raw")
dataDZOF <- mxData(observed = DZOF_data, type = "raw")
dataDZOM <- mxData(observed = DZOM_data, type = "raw")

mz <- rbind(MZF_data, MZM_data)
dz <- rbind(DZF_data, DZM_data, DZO_data)

dataMZ <- mxData(observed = mz, type = "raw")
dataDZ <- mxData(observed = dz, type = "raw")


#FULL ACE CHOLESKY - MATRIX####


#Need helper functions:
http://ibg.colorado.edu/cdrom2014/bartels/Multivariate/GenEpiHelperFunctions.R
#http://ibg.colorado.edu/cdrom2014/bartels/Multivariate/myFunctions.R

nv=6
svPa      <- valDiag(nv,.6)               # start values for parameters on diagonal
lbPa      <- valLUDiag(nv,.0001,-10,NA)   # lower bounds for parameters on diagonal


pathA_chol     <- mxMatrix( type="Lower", nrow=nv, ncol=nv, free=TRUE,
                            values = c(8, 13.8, .7, .76, .56, .98,
                                       13.8, .7, .76, .56, .98,
                                       .7, .76, .56, .98,
                                       .76, .56, .98,
                                       .56, .98,
                                       .98), labels=labLower("a",nv), lbound=lbPa, name="a" )
pathC_chol     <- mxMatrix( type="Lower", nrow=nv, ncol=nv, free=TRUE,
                            values = c(8, 13.8, .7, .76, .56, .98,
                                       13.8, .7, .76, .56, .98,
                                       .7, .76, .56, .98,
                                       .76, .56, .98,
                                       .56, .98,
                                       .98), labels=labLower("c",nv), lbound=lbPa, name="c" )
pathE_chol     <- mxMatrix( type="Lower", nrow=nv, ncol=nv, free=TRUE,
                            values = c(8, 13.8, .7, .76, .56, .98,
                                       13.8, .7, .76, .56, .98,
                                       .7, .76, .56, .98,
                                       .76, .56, .98,
                                       .56, .98,
                                       .98), labels=labLower("e",nv), lbound=lbPa, name="e" )

# Matrices generated to hold A, C, and E computed Variance Components
covA_chol      <- mxAlgebra( expression=a %*% t(a), name="A" )
covC_chol      <- mxAlgebra( expression=c %*% t(c), name="C" )
covE_chol      <- mxAlgebra( expression=e %*% t(e), name="E" )

# Algebra to compute total variances and standard deviations (diagonal only)
covP_chol      <- mxAlgebra( expression=A+C+E, name="V" )
matI_chol      <- mxMatrix( type="Iden", nrow=nv, ncol=nv, name="I")
invSD_chol     <- mxAlgebra( expression=solve(sqrt(I*V)), name="iSD")


meansF <- mxMatrix(type = "Full", nrow = 1, ncol = 12, free = T, values = c(108, 109, 1.52,
0.22, 0.29, -0.01, 108, 109, 1.52, 0.22, 0.29, -0.01),
                   label = c("VIQF", "PIQF", "RD", "CC", "CPF", "ATPF", "VIQF", "PIQF",
```

```
                        "RD", "CC", "CPF", "ATPF"), name = "meanF")
meansM <- mxMatrix(type = "Full", nrow = 1, ncol = 12, free = T, values = c(112, 114,
1.52,0.22, 0.06, 0.43, 112, 114, 1.52,0.22, 0.06, 0.43),
                        label = c("VIQM", "PIQM", "RD", "CC", "CPM", "ATPM", "VIQM", "PIQM",
                        "RD", "CC", "CPM", "ATPM"), name = "meanM")
meansFM <- mxMatrix(type = "Full", nrow = 1, ncol = 12, free = T, values = c(108, 109, 1.52,
0.22, 0.29, -0.01, 112, 114, 1.52,0.22, 0.06, 0.43),
                         label = c("VIQF", "PIQF", "RD", "CC", "CPF", "ATPF", "VIQM", "PIQM",
                         "RD", "CC", "CPM", "ATPM"), name = "meanFM")
meansMF <- mxMatrix(type = "Full", nrow = 1, ncol = 12, free = T, values = c(112, 114,
1.52,0.22, 0.06, 0.43,108, 109, 1.52, 0.22, 0.29, -0.01),
                         label = c("VIQM", "PIQM", "RD", "CC", "CPM", "ATPM", "VIQF", "PIQF",
                         "RD", "CC", "CPF", "ATPF"), name = "meanMF")

covMZ_chol      <- mxAlgebra(expression= rbind( cbind(V, A+C), cbind(A+C, V)),
name="expCovMZ_chol" )
covDZ_chol      <- mxAlgebra(expression= rbind( cbind(V, 0.5%x%A+C), cbind(0.5%x%A+C, V)),
name="expCovDZ_chol" )

expMZF      <- mxExpectationNormal( covariance="expCovMZ_chol", means= "meanF",
dimnames=selVars )
expMZM      <- mxExpectationNormal( covariance="expCovMZ_chol", means= "meanM",
dimnames=selVars )
expDZF      <- mxExpectationNormal( covariance="expCovDZ_chol", means="meanF",
dimnames=selVars )
expDZM      <- mxExpectationNormal( covariance="expCovDZ_chol", means="meanM",
dimnames=selVars )
expDZFM      <- mxExpectationNormal( covariance="expCovDZ_chol", means="meanFM",
dimnames=selVars )
expDZMF      <- mxExpectationNormal( covariance="expCovDZ_chol", means="meanMF",
dimnames=selVars )

pars_chol       <- list( pathA_chol, pathC_chol, pathE_chol, covA_chol, covC_chol, covE_chol,
covP_chol, matI_chol, invSD_chol)

funML      <- mxFitFunctionML()

ACE_modelMZF    <- mxModel( name="ACE_MZF", meansF, pars_chol, covMZ_chol, dataMZF, funML,
expMZF )
ACE_modelMZM    <- mxModel( name="ACE_MZM", meansM, pars_chol, covMZ_chol, dataMZM, funML,
expMZM )
ACE_modelDZF    <- mxModel( name="ACE_DZF", meansF, pars_chol, covDZ_chol, dataDZF, expDZF,
funML )
ACE_modelDZM    <- mxModel( name="ACE_DZM", meansM, pars_chol, covDZ_chol, dataDZM, expDZM,
funML )
ACE_modelDZFM    <- mxModel( name="ACE_DZFM", meansFM, pars_chol, covDZ_chol, dataDZOF,
expDZFM, funML )
ACE_modelDZMF    <- mxModel( name="ACE_DZMF", meansMF, pars_chol, covDZ_chol, dataDZOM,
expDZMF, funML )

multi      <- mxFitFunctionMultigroup( c("ACE_MZF", "ACE_MZM", "ACE_DZF", "ACE_DZM",
"ACE_DZFM", "ACE_DZMF" ) )

a_std <- mxAlgebra(expression = iSD %*% a, name = "std_a")
c_std <- mxAlgebra(iSD %*% c, name = "std_c")
e_std <- mxAlgebra(iSD %*% e, name = "std_e")
CI_std <- mxCI(c("std_a", "std_c", "std_e" ))

ACE_model <- mxModel("CholACE", pars_chol, ACE_modelMZF, ACE_modelMZM, ACE_modelDZF,
                     ACE_modelDZM, ACE_modelDZFM, ACE_modelDZMF, multi, a_std, c_std, e_std,
                     CI_std)
ACE_fit <- mxRun(ACE_model, intervals = F)
summary(ACE_fit)

#Cholesky w. Dropped Paths####

ACE_simp_1 <- omxSetParameters(ACE_model, labels = c("a_6_5", "a_6_4", "a_5_4",  "a_5_3",
"a_4_3",  "a_3_2", "a_4_2","a_5_2",
```

```
                                                "c_6_5", "c_6_4", "c_5_4", "c_6_3",
                                                "c_5_3", "c_4_3", "c_6_2", "c_4_2",
                                                "c_3_2", "c_5_2",
                                                "e_4_1", "e_3_2", "e_5_2", "e_5_4",
                                                "e_6_5","e_5_1", "a_4_4", "c_4_4",
                                                "c_3_3", "c_2_2",
                                                "c_6_6", "e_4_3", "e_6_3", "c_4_1",
                                                "a_5_5", "c_5_5"),
                                free = F, values = 0)
ACE_simp_1 <- mxRun(ACE_simp_1, intervals = F)
summary(ACE_simp_1)
mxCompare(ACE_fit, ACE_simp_1)  #no sig. loss

ACE_simp_1 <- mxRun(ACE_simp_1, intervals = T)

# ACE Squared Standardized Path Coefficients
a2_simp <- mxAlgebra(((iSD%*% a)*(iSD%*% a)), name = "a2_simp")
c2_simp <- mxAlgebra((iSD%*% c)*(iSD%*% c), name = "c2_simp")
e2_simp <- mxAlgebra((iSD%*% e)*(iSD%*% e), name = "e2_simp")
a2_simp_sum <- mxAlgebra(rowSums(((iSD%*% a)*(iSD%*% a))), name = "a2_sum")
c2_simp_sum <- mxAlgebra(rowSums(((iSD%*% c)*(iSD%*% c))), name = "c2_sum")
e2_simp_sum <- mxAlgebra(rowSums((iSD%*% e)*(iSD%*% e)), name = "e2_sum")

# proportion of phenotypic correlation due to genetic effects
rph_a <- mxAlgebra(A/V, name = "rph_a")
rph_c <- mxAlgebra(C/V, name = "rph_c")
rph_e <- mxAlgebra(E/V, name = "rph_e")

corA      <- mxAlgebra( expression=solve(sqrt(I*A))%&%A, name ="rA" ) #cov2cor()
corC      <- mxAlgebra( expression=solve(sqrt(I*C))%&%C, name ="rC" )
corE      <- mxAlgebra( expression=solve(sqrt(I*E))%&%E, name ="rE" )
corP <- mxAlgebra(expression= solve(sqrt(I*V)) %*% V %*% solve(sqrt(I*V)), name="rPH")

ACE_simp_final <- mxModel(ACE_simp_1, name = "final_simp", mxCI(c("rA", "rE", "rPH",
"a2_simp", "c2_simp", "e2_simp", "a2_sum", "e2_sum", "c2_sum", "rph_a", "rph_c", "rph_e")),
                          a2_simp, c2_simp, e2_simp, a2_simp_sum, e2_simp_sum, c2_simp_sum,
                          rph_a, rph_c, rph_e, corA, corC, corE, corP)
ACE_simp_final <- mxRun(ACE_simp_final, intervals = T)

write.csv(ACE_simp_final$output$confidenceIntervals, 'simpCI.csv')

# Generate List of Parameter Estimates and Derived Quantities using formatOutputMatrices
IndACEpathMatrices <- c("iSD %*% ac","iSD %*% cc","iSD %*% ec","iSD %*% as")
IndACEpathLabels <- c("stPathAc","stPathCc","stPathEc","stPathAs")
formatOutputMatrices(drop_fitAs,IndACEpathMatrices,IndACEpathLabels,selVars_1,6)


# TEST OF HYPOTHESISED MODEL

nf <- 2
nv <- 6
ntv <- 12
svPa <- valDiag(nv, .6)
lbPa      <- valLUDiag(nv,.0001,-10,NA)

# Matrices ac, cc, and ec to store a, c, and e path coefficients for common factors
pathAc    <- mxMatrix( type="Full", nrow=nv, ncol=nv, free=c(T, T, T, T, T, T,
                                                              F, T, F, F, F, T,
                                                              F, F, T, T, T, T,
                                                              F, F, F, F, F, F,
                                                              F, F, F, F, F, F,
                                                              F, F, F, F, F, F),
                       values=c(8, 13.8, .7, .76, .56, .98,
                                0, 7, 0, 0, 0, .98,
                                0, 0, .7, .76, .56, .98,
                                0,0,0,0,0,0,
                                0,0,0,0,0,0,
                                0,0,0,0,0,0), labels=labFull("ac",nv,nv), name="ac",
```

```
                       lbound = c(.0000001, .0000001, .0000001, .0000001, .0000001, .0000001,
                                  0, .0000001, 0, 0, 0, .0000001,
                                  0, 0, -10, -10, -10, -10,
                                  0,0,0,0,0,0,
                                  0,0,0,0,0,0,
                                  0,0,0,0,0,0))

pathCc    <- mxMatrix( type="Full", nrow=nv, ncol=1, free= TRUE,
                       values=c(8, 13.8, .7, .76, .56, .98), labels=labFull("cc",nv,1),
                       name="cc" )


pathEc     <- mxMatrix(type="Lower", nrow=nv, ncol=nv, free=TRUE, values = c(8, 13.8, .7,
.76, .56, .98,
                                                                             13.8, .7, .76,
                                                                             .56, .98,
                                                                             .7, .76, .56,
                                                                             .98,
                                                                             .76, .56, .98,
                                                                             .56, .98,
                                                                             .98),
                                                                             label=labLower("e
                                                                             c",nv),
                                                                             lbound=lbPa,
                                                                             name="ec" )



# Matrix as to store a path coefficients for specific factors
pathAs    <- mxMatrix( type="Diag", nrow=nv, ncol=nv, free=c(T, T, F, T, T, T),
                       values=c(8, 13.8, .7, .76, .56, .98), labels=labDiag("as",nv),
                       name="as" )

covA      <- mxAlgebra( expression=ac %*% t(ac) + as %*% t(as), name="A" )
covC      <- mxAlgebra( expression=cc %*% t(cc), name="C" )
covE      <- mxAlgebra( expression=ec %*% t(ec), name="E" )

covP      <- mxAlgebra( expression= A+C+E, name="V" )
covMZ     <- mxAlgebra( expression= rbind( cbind(V, A+C), cbind(A+C, V)), name="expCovMZ" )
covDZ     <- mxAlgebra( expression= rbind( cbind(V, 0.5%x%A+C), cbind(0.5%x%A+C, V)),
name="expCovDZ" )


matI      <- mxMatrix( type="Iden", nrow=6, ncol=6, name="I")
invSD     <- mxAlgebra( expression=solve(sqrt(I*V)), name="iSD")

corA      <- mxAlgebra( expression=solve(sqrt(I*A))%&%A, name ="rA" ) #cov2cor()
corC      <- mxAlgebra( expression=solve(sqrt(I*C))%&%C, name ="rC" )
corE      <- mxAlgebra( expression=solve(sqrt(I*E))%&%E, name ="rE" )
corP <- mxAlgebra(expression= solve(sqrt(I*V)) %*% V %*% solve(sqrt(I*V)), name="rPH")

meansF <- mxMatrix(type = "Full", nrow = 1, ncol = 12, free = T, values = c(108, 109, 1.52,
0.22, 0.29, -0.01, 108, 109, 1.52, 0.22, 0.29, -0.01),
                   label = c("VIQF", "PIQF", "RD", "CC", "CPF", "ATPF", "VIQF", "PIQF",
                   "RD", "CC", "CPF", "ATPF"), name = "meanF")
meansM <- mxMatrix(type = "Full", nrow = 1, ncol = 12, free = T, values = c(112, 114,
1.52,0.22, 0.06, 0.43, 112, 114, 1.52,0.22, 0.06, 0.43),
                   label = c("VIQM", "PIQM", "RD", "CC", "CPM", "ATPM", "VIQM", "PIQM",
                   "RD", "CC", "CPM", "ATPM"), name = "meanM")
meansFM <- mxMatrix(type = "Full", nrow = 1, ncol = 12, free = T, values = c(108, 109, 1.52,
0.22, 0.29, -0.01, 112, 114, 1.52,0.22, 0.06, 0.43),
                    label = c("VIQF", "PIQF", "RD", "CC", "CPF", "ATPF", "VIQM", "PIQM",
                    "RD", "CC", "CPM", "ATPM"), name = "meanFM")
meansMF <- mxMatrix(type = "Full", nrow = 1, ncol = 12, free = T, values = c(112, 114,
1.52,0.22, 0.06, 0.43,108, 109, 1.52, 0.22, 0.29, -0.01),
                    label = c("VIQM", "PIQM", "RD", "CC", "CPM", "ATPM", "VIQF", "PIQF",
                    "RD", "CC", "CPF", "ATPF"), name = "meanMF")
```

```
expMZF     <- mxExpectationNormal( covariance="expCovMZ", means= "meanF", dimnames=selVars )
expMZM     <- mxExpectationNormal( covariance="expCovMZ", means= "meanM", dimnames=selVars )
expDZF     <- mxExpectationNormal( covariance="expCovDZ", means="meanF", dimnames=selVars )
expDZM     <- mxExpectationNormal( covariance="expCovDZ", means="meanM", dimnames=selVars )
expDZFM     <- mxExpectationNormal( covariance="expCovDZ", means="meanFM", dimnames=selVars )
expDZMF     <- mxExpectationNormal( covariance="expCovDZ", means="meanMF", dimnames=selVars )

pars       <- list( pathAc, pathCc, pathEc, pathAs,
                   covA, covC, covE, covP, matI, invSD, corA, corC, corE, corP )
funML      <- mxFitFunctionML()

stip_modelMZF   <- mxModel( name="stip_MZF", meansF, pars, covMZ, dataMZF, funML, expMZF )
stip_modelMZM   <- mxModel( name="stip_MZM", meansM, pars, covMZ, dataMZM, funML, expMZM )
stip_modelDZF   <- mxModel( name="stip_DZF", meansF, pars, covDZ, dataDZF, expDZF, funML )
stip_modelDZM   <- mxModel( name="stip_DZM", meansM, pars, covDZ, dataDZM, expDZM, funML )
stip_modelDZFM   <- mxModel( name="stip_DZFM", meansFM, pars, covDZ, dataDZOF, expDZFM,
funML )
stip_modelDZMF   <- mxModel( name="stip_DZMF", meansMF, pars, covDZ,dataDZOM, expDZMF, funML )

ac_std <- mxAlgebra(expression = iSD %*% ac, name = "std_ac")
cc_std <- mxAlgebra(iSD %*% cc, name = "std_cc")
ec_std <- mxAlgebra(iSD %*% ec, name = "std_ec")
as_std <- mxAlgebra(iSD %*% as, name = "std_as")
CI_std <- mxCI(c("std_ac", "std_ec","std_cc", "std_as" ))

multi      <- mxFitFunctionMultigroup( c("stip_MZF", "stip_MZM", "stip_DZF", "stip_DZM",
"stip_DZFM", "stip_DZMF" ) )
stip_modelACE <- mxModel(name = "stip_ACE", pars, stip_modelMZF, stip_modelMZM,
                         stip_modelDZF, stip_modelDZM, stip_modelDZFM, stip_modelDZMF, multi,
                         ac_std, cc_std, ec_std, as_std, CI_std)

stip_fitACE <- mxRun(stip_modelACE, intervals = F)
summary(stip_fitACE)

mxCompare(ACE_fit, stip_fitACE)
```
